# Protein functional links in *Trypanosoma brucei*, identified by gene fusion analysis

**DOI:** 10.1186/1471-2148-11-193

**Published:** 2011-07-05

**Authors:** Dimitris Dimitriadis, V Lila Koumandou, Philip Trimpalis, Sophia Kossida

**Affiliations:** 1 Biomedical Research Foundation, Academy of Athens, Athens, Greece

## Abstract

**Background:**

Domain or gene fusion analysis is a bioinformatics method for detecting gene fusions in one organism by comparing its genome to that of other organisms. The occurrence of gene fusions suggests that the two original genes that participated in the fusion are functionally linked, i.e. their gene products interact either as part of a multi-subunit protein complex, or in a metabolic pathway. Gene fusion analysis has been used to identify protein functional links in prokaryotes as well as in eukaryotic model organisms, such as yeast and *Drosophila*.

**Results:**

In this study we have extended this approach to include a number of recently sequenced protists, four of which are pathogenic, to identify fusion linked proteins in *Trypanosoma brucei*, the causative agent of African sleeping sickness. We have also examined the evolution of the gene fusion events identified, to determine whether they can be attributed to fusion or fission, by looking at the conservation of the fused genes and of the individual component genes across the major eukaryotic and prokaryotic lineages. We find relatively limited occurrence of gene fusions/fissions within the protist lineages examined. Our results point to two trypanosome-specific gene fissions, which have recently been experimentally confirmed, one fusion involving proteins involved in the same metabolic pathway, as well as two novel putative functional links between fusion-linked protein pairs.

**Conclusions:**

This is the first study of protein functional links in *T. brucei *identified by gene fusion analysis. We have used strict thresholds and only discuss results which are highly likely to be genuine and which either have already been or can be experimentally verified. We discuss the possible impact of the identification of these novel putative protein-protein interactions, to the development of new trypanosome therapeutic drugs.

## Background

Proteins exert their diverse functions usually through interactions with other molecules, and indeed often through interactions with other proteins. Protein-protein interactions are currently the focus of intensive study, as they allow functional characterisation of proteins and help interpret the role of uncharacterised gene products in organisms with fully sequenced genomes. Apart from direct biochemical analysis to detect protein-protein interactions, an *in silico *approach is sometimes used to predict protein-protein interactions. This fusion analysis (also known as "Rosetta stone" method) takes advantage of the study of genomic structures and sequence similarity to detect putative interacting protein pairs, which, importantly might not have been suspected based on current biochemical knowledge. Briefly, if a pair of non-homologous proteins which are found in different genomic regions in organism A, are found fused into a single ORF in organism B, this suggests that the two independent proteins in organism A may interact. These protein-protein interactions may be transient or more long-lived, either within a metabolic pathway, or as part of a multi-subunit protein complex.

Fusion analysis has been used to identify putative protein-protein interactions in completely sequenced genomes of various prokaryotes, and eukaryotes [1-13]. Here we have applied gene fusion analysis to a number of recently sequenced protists, and in particular tried to infer interacting protein pairs in the pathogenic parasite *Trypanosoma brucei. Trypanosome brucei*, a flagellated parasitic protist, is responsible for African trypanosomiasis, a neglected disease known as sleeping sickness in humans. The protozoan uses the tsetse fly as an insect vector, and the sickness is fatal if untreated. All the available treatment drugs are generally unsatisfactory, due to dangerous side effects, high cost of production, and difficulty in distribution [14]. Protein-protein interaction analysis can thus provide clues into new protein functions and new potential drug targets. Importantly, *T. brucei *is amenable to genetic manipulation for verification of the results presented here.

We chose seven other organisms for our analysis, representing all major eukaryotic lineages (Table 1). Four of the organisms analysed are also pathogenic:

**Table 1:** List of organisms used in this study.

Kingdom	Phylum	Family	Species	Category	Disease
**Excavata**	Metamonada	Hexamitidae	*Giardia intestinalis*	Pathogenic organism	Giardiasis

**Unikonta**	Amoebozoa	Amoebida	*Entamoeba histolytica*	Pathogenic organism	Amoebic dysentery
	
	Ascomycota	Saccharomycetaceae	*Candida albicans*	Pathogenic organism	Candidiasis
	
	Metazoa	Codonosigidae	*Monosiga brevicollis*	Model organism	
	
	Mycetozoa	Dictyosteliidae	*Dictyostelium discoideum*	Model organism	

**Chromalveolata**	Heterokontophyta	Pythiaceae	*Phytophthora infestans*	Pathogenic organism	Late potato blight

**Plantae**	Chlorophyta	Chlamydomoadaceae	*Chlamydomonas reinhardtii*	Model organism	

The proteomes of the species studied were downloaded as FASTA files from the NCBI or UniProt databases and used during the domain fusion analysis as target organisms against *Trypanosoma brucei*.

(1) *Giardia intestinalis*, another flagellated parasite in the excavate group, is a common pathogen infecting not only humans, but also dogs, birds and cats. This single-celled organism is responsible for giardiasis, an infection of the small intestine which causes diarrhea and which may be fatal for people with a compromised immune system [15].

(2) *Candida albicans *is a diploid fungus, residing in the human mouth and gastrointestinal tract. Under normal conditions, the microorganism does not affect humans. However, under certain circumstances, the overgrowth of *C. albicans *results in candidiasis, also known as "thrush", which occurs in the blood and genital track and mainly affects people with a compromised immune system [16].

(3) *Entamoeba histolytica *is an amoeba that infects mammals, such as dogs and cats along with human beings. *Entamoeba *infection leads to amoebic dysentery and liver abscess, including fulminating dysentery, bloody diarrhea, weight loss, fatigue, abdominal pain, and amoeboma [17].

(4) *Phytophthora infestans*, an oomycete that affects agricultural crops, is responsible for the late blight potato disease, also known as potato blight. The organism can also infect tomatoes [18].

Three model organisms were also used in this study:

(5) *Dictyostelium discoideum*, an amoeba with an asexual life cycle, is a natural host for many bacteria, some digested and some not, which lead to the death of the host after their proliferation. It might constitute a role model organism, to identify how macrophage defends itself against intruders [19,20].

(6) *Chlamydomonas reinhardtii*, a single celled green alga is a model organism mainly used to study cell mobility, how the cells regulate their proteome to control flagellar length, and how cells respond to changes in mineral nutrition. In addition, *Chlamydomonas reinhardtii *attracts great interest due to its ability to photosynthetically produce molecular hydrogen [21,22].

(7) *Monosiga brevicollis*, a unicellular flagellated organism, is part of the Choanoflagellates which are aquatic protists and the closest known relatives of metazoans [23].

The automatic analysis identified putative gene fusions in all organisms, but we discuss only those in *Entamoeba, Phytophthora, Chlamydomonas *and *Monosiga*, which passed all further verification steps. Two of the results have previously been experimentally verified.

## Results and Discussion

To determine protein linkages between a kinetoplastid protozoan species, *Trypanosoma brucei*, and seven protists representing all major eukaryotic lineages, we used the domain fusion analysis method modified by our in-house software as described in the methods. *Trypanosoma brucei TREU927 *was used as a reference organism and its complete proteome of 8788 proteins was compared with the proteomes of the seven target species (Table 1), in terms of domain architecture, to identify distant relationships. This analysis gave us a total of 81 putative composites (Table 2). Furthermore, within the set of 81 domain architectures, we tried to verify which represented proteins that evolved by fusion. The criteria for verifying the predicted events included best hit of the reverse blast process, blast e-value threshold parameter, length of composite protein, length of fused protein, similarities of function between split and composite domain architectures and their role in biological pathways. Any result which did not accord with these criteria was not analysed further. Only 12 composites passed all the verification steps (Table 2). Furthermore, after checking for conservation in the closely related species *Leishmania major*, only 5 composites were considered genuine. We discuss the final results, representing functional linkages, separately for each species.

**Table 2:** Summary of results from the domain fusion analysis.

			Fusion		
			
Species	Proteome size	Total	Verified	Potential Genuine	EMBL Bank Accession
*Giardia intestinalis*	7,147	3	2		

***Entamoeba histolytica***	7,948	38	1	1	EAL47672

*Candida albicans*	5,782	7	1		

***Monosiga brevicolis***	9,156	9	2	1	EDQ88211

*Dictyostelium discoideum*	12,903	3	2		

***Phytophthora infestans***	18,264	15	2	1	EEY58132

***Chlamydomonas reinhardtii***	15,067	6	2	2*	EDP05938 &EDP08267

For each species studied, the full number of proteins in the proteome is given, as well as the number of fusion events identified after the automatic analysis (Total), after manual verification including reverse BLAST score and domain annotations (Verified), and after a further verification of the results in the closely related species *Leishmania major *(Potential Genuine). Asterisks denote results that have already been experimentally verified. EMBL accession numbers for the final results discussed in the text are also given, and the organisms that gave potential genuine results are highlighted in bold.

The number of interactions identified is lower than might be expected, but it should be noted that our method is highly selective. We tested the performance of our automatic in-house software by comparing the same organisms analysed by Enright *et al. *[2] and we detected almost 90% of the events reported by Enright *et al. *(Additional file 1). However, only 20% of the events reported by Enright *et al. *conform to all our selection criteria (%coverage, similarity, etc.) although some novel events were also detected by our program which were not reported by Enright *et al. *(Additional file 1).

### Example of fusion links in *Phytophthora infestans *(EEY58132)

**Figure 1:**
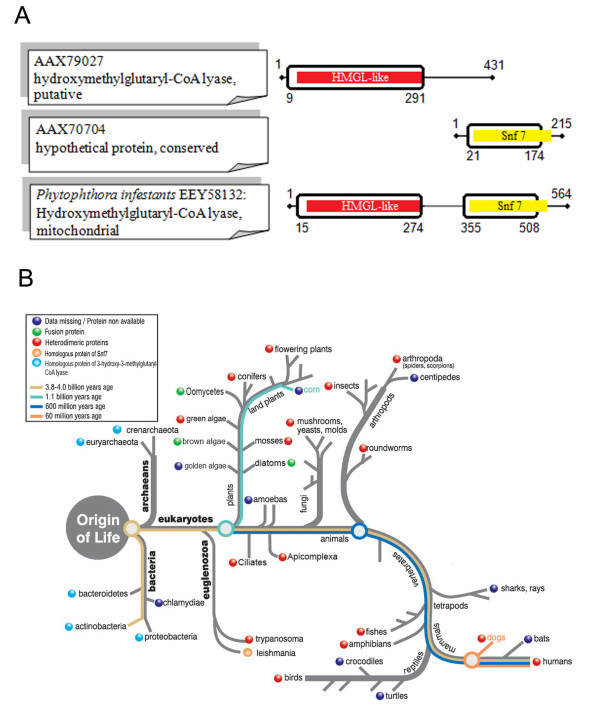
**Fusion linked result for *Phytopthora infestans***. (A) Schematic alignment of the *T. brucei *protein pair with the fused protein in *P. infestans*, showing the amino acid positions that delineate the beginning and end of the alignment, as well as the position of the conserved domains, relative to the full protein length. For the full details of the alignment see Additional file 2. (B) Fusion analysis for the specific protein pair across the range of eukaryotes and prokaryotes, mapped on a generic evolutionary tree, to highlight the evolution of the fusion event.

From the automatic analysis, we found 15 predicted fusion links between pairs of proteins in *P. infestans *and *T. brucei*. Following the more detailed analysis, including reverse BLAST, only one of these, EEY58132, conformed to all our selection criteria. The domain architecture of the fusion linked protein identified in *Phytophthora infestans *corresponds to the domain architecture of the corresponding split protein pair in the *T. brucei *proteome. The *T. brucei *proteins AAX79027 and AAX70704 are non-homologous, reside on different chromosomes, and are annotated as "putative hydroxymethylglutaryl-CoAlyase" and "conserved hypothetical", respectively. The region of alignment between EEY58132 and AAX79027 extends for 282 amino acids, and coincides with the HMGL-Like family domain (PF00682) which is found in a diverse set of enzymes, including various aldolases and pyruvate carboxylase (Figure 1A). The region of alignment between EEY58132 and AAX70704 extends for 153 amino acids and coincides with the Snf7 domain (PF03357) which is found in a family of proteins that are involved in protein sorting and transport from the endosome to the vacuole or lysosome in eukaryotic cells (Figure 1A for full details of the alignment see Additional file 2). As is often seen for modular proteins, independent domains of two proteins might be found adjacent to each other in other proteins. Thus, a link between a protein associated with a HMG-CoA lyase deficiency and a protein with a Snf7 functional domain, suggests that they both function within the same pathway. This is unexpected, as AAX79027 (Tb927.4.2700) appears to be a true hydroxymethylglutaryl-CoAlyase orthologue by reverse BLAST. This enzyme (EC 4.1.3.4) is involved in leucine metabolism and ketogenesis and localises to mitochondria and peroxisomes in humans [24]. In *T. brucei*, this proteins was recently predicted to localise to mitochondria [25]. In contrast, AAX70704 (Tb927.8.5430) has been reported as a Vps20 orthologue, a component of ESCRTIII, which is involved in sorting ubiquitylated proteins at the multi-vesicular-body for degradation in the lysosome [26]. Thus an association between these two proteins in *Phytophthora *(and other organisms, see Figure 1B), might suggest a role for ubiquitylation in the sorting of hydroxymethylglutaryl-CoAlyase.

When we examine the evolution of the fusion linked protein pair across the full range of eukaryotes and prokaryotes, the data suggest multiple independent fusion events within the archaeplastida (in diatoms, brown algae, and oomycetes Figure 1B). Only one of the two proteins of the composite is conserved in prokaryotes (Figure 1B), namely the hydroxymethylglutaryl-CoAlyase, since prokaryotes do not posses the full ubiquitylation machinery. Although divergent ESCRTIII factors have been identified recently [27], these are not readily detectable by BLAST. Surprisingly, only the Snf7 part is conserved in *Leishmania*, while the hydroxymethylglutaryl-CoAlyase enzyme seems to be missing ([14], see supplementary table ten). It is thought that in *L. major *HMGCoA is incorporated directly into sterols by the isoprenoid synthetic pathway [28].

### Example of fusion links in *Monosiga brevicolis *(EDQ88211)

**Figure 2:**
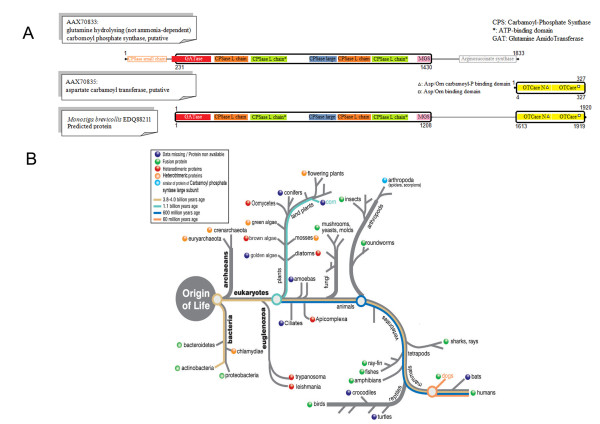
**Fusion linked result for *Monosiga brevicollis***. (A) Schematic alignment of the *T. brucei *protein pair with the fused protein in *M. brevicollis*, showing the amino acid positions that delineate the beginning and end of the alignment, as well as the position of the conserved domains, relative to the full protein length. For the full details of the alignment see Additional file 2. (B) Fusion analysis for the specific protein pair across the range of eukaryotes and prokaryotes, mapped on a generic evolutionary tree, to highlight the evolution of the fusion event.

From the automatic analysis, we found 9 predicted fusion links between pairs of proteins in *M. brevicollis *and *T. brucei*. Following the more detailed analysis, including reverse BLAST, only one of these, EDQ88211, conformed to all our selection criteria. The domain architecture of the fusion linked protein identified in *Monosiga brevicolis *corresponds to the domain architecture of the corresponding split protein pair in the *T. brucei *proteome. The *T. brucei *non-homologous proteins, AAX70833 and AAX70835, are annotated as "putative glutamine hydrolysing (not ammonia-dependent) carbamoyl phosphate synthase" (EC 6.3.5.5) and "putative aspartate carbamoyltransferase" (EC 2.1.3.2), respectively. The region of alignment between EDQ88211 and AAX70833 extends for 1199 amino acids, and coincides with the carbamoyl-phosphate synthase L-chain & glutamine amidotransferase class-I domains (PF00289 & PF00117) (Figure 2A). The region of alignment between EDQ88211 and AAX70835 extends for 323 amino acids, and coincides with the OTCase_N & OTCase domains (PF02729 & PF00185) which catalyze the conversion of ornithine and carbomoyl phosphate to citrulline (Figure 2A). A link between these proteins suggests that they both function within the same pathway. Indeed, they are both involved in pyrimidine biosynthesis and catalyse two subsequent reaction steps in the pathway [29,30]. Interestingly, the two *T. brucei *genes encoding these proteins (Tb927.5.3800 and Tb927.5.3820, respectively) reside on the same chromosome, interspersed by a single ORF. The intervening ORF (Tb927.5.3810) is annotated as "putative orotidine-5-phosphate decarboxylase/orotate phosphoribosyltransferase" (EC 4.1.1.23 and EC 2.4.2.10, respectively), which also participates in the same pathway, at a later step. Given that the proteins participate in the same pathway, it is perhaps not surprising that the fusion seems to have occurred before the divergence of the fungi and metazoa and also within some branches of the eubacteria (Figure 2B). Indeed, where the protein pair is not found as a fused composite, it is often heterotrimeric, suggesting further fission of one of the multi-domain components (Figure 2B).

### Example of fusion links in *Entamoeba histolytica *(EAL47672)

**Figure 3:**
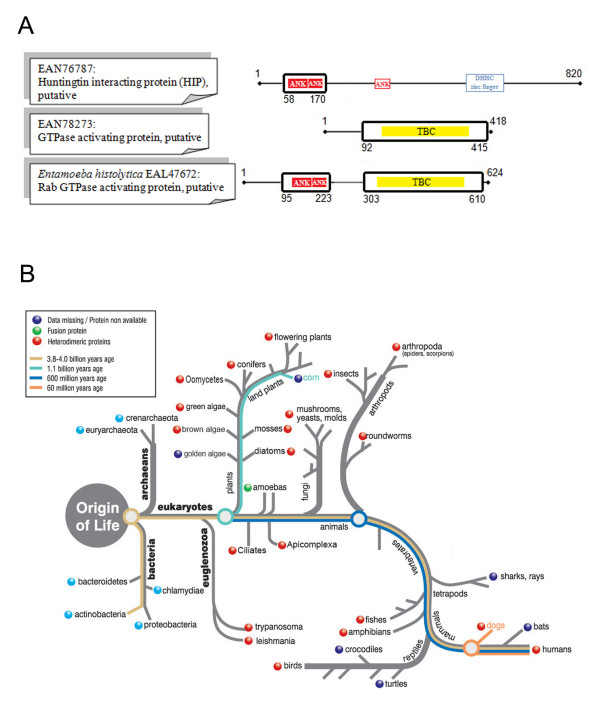
**Fusion linked result for *Entamoeba histolytica***. (A) Schematic alignment of the *T. brucei *protein pair with the fused protein in *E. histolytica*, showing the amino acid positions that delineate the beginning and end of the alignment, as well as the position of the conserved domains, relative to the full protein length. For the full details of the alignment see Additional file 2. (B) Fusion analysis for the specific protein pair across the range of eukaryotes and prokaryotes, mapped on a generic evolutionary tree, to highlight the evolution of the fusion event.

From the automatic analysis, we found 38 predicted fusion links between pairs of proteins in *E. histolytica *and *T. brucei*. Following the more detailed analysis, only one of these, EAL47672, conformed to all our selection criteria. The domain architecture of the fusion linked protein identified in *Entamoeba histolytica *corresponds to the domain architecture of the corresponding split protein pair in the *T. brucei *proteome. The *T. brucei *proteins EAN76787 and EAN78273 are non-homologous, reside on different chromosomes, and are annotated as "putative huntingtin interacting protein" and "putative GTPase activating protein", respectively. The region of alignment between EAL47672 and EAN76787 extends for 112 amino acids, and coincides with two Ankyrin repeat domains (PF00023) (Figure 3A). Huntingtin interacting proteins regulate the cytotoxicity of Huntingtin, a protein whose mutation causes Huntington's disease [31,32]. However, it is unclear why the *T. brucei *protein has been annotated as "putative huntingtin interacting protein", as it does not contain any of the expected conserved domains for such a function, and does not match huntingtin interacting proteins when checked by reverse BLAST. Instead, all the top reverse BLAST hits are characterised by the presence of the Ankyrin repeats, which mediate protein-protein interactions. The region of alignment between EAL47672 and EAN78273 extends for 323 amino acids, and coincides with the TBC domain (PF00566) which is found in GTPase activator proteins of Rab-like small GTPases (Figure 3A). Thus, a link between a protein with ANK domains and a protein with a GTPase activating functional domain, suggests that they both function within the same pathway. Indeed, this agrees with a recent hypothesis proposed by Kanno *et al. *that TBC domain proteins do not directly interact with Rabs, but depend on other protein-protein interaction domains, such as ankyrin repeats; this was shown by studying the binding of truncation mutants for a protein containing both a Rab-GAP and a C-terminal ANK domain [33]. Analysis of the evolution of this fusion linked protein pair indicates that the fusion detected is unique to the amoebozoa, while prokaryotes do not posses TBC domains (Figure 3B).

### Example of fusion links in *Chlamydomonas reinhardtii *(EDP05938)

From the automatic analysis, we found 6 predicted fusion links between pairs of proteins in the two species. Following the more detailed analysis, only two of these, EDP05938 & EDP08267, conformed to all our selection criteria. The domain architecture of the fusion linked proteins identified in *Chlamydomonas reinhardtii *correspond to the domain architecture of the corresponding split proteins pair in the *T. brucei *proteome.

**Figure 4:**
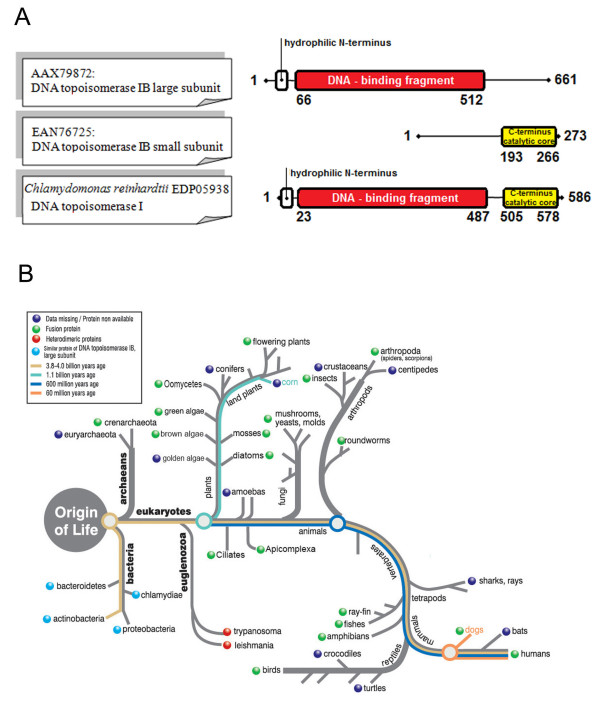
**Fusion linked result for *Chlamydomonas reinhardtii *topoisomerase**. (A) Schematic alignment of the *T. brucei *protein pair with the fused protein in *C. reinhardtii*, showing the amino acid positions that delineate the beginning and end of the alignment, as well as the position of the conserved domains, relative to the full protein length. For the full details of the alignment see Additional file 2. (B) Fusion analysis for the specific protein pair across the range of eukaryotes and prokaryotes, mapped on a generic evolutionary tree, to highlight the evolution of the fusion event.

**Figure 5:**
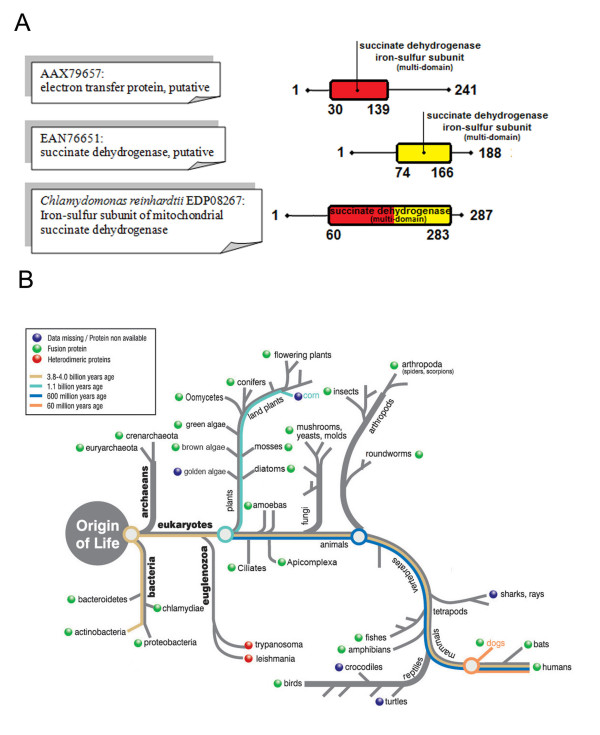
**Fusion linked result for *Chlamydomonas reinhardtii *succinate dehydrogenase**. (A) Schematic alignment of the *T. brucei *protein pair with the fused protein in *C. reinhardtii*, showing the amino acid positions that delineate the beginning and end of the alignment, as well as the position of the conserved domains, relative to the full protein length. For the full details of the alignment see Additional file 2. (B) Fusion analysis for the specific protein pair across the range of eukaryotes and prokaryotes, mapped on a generic evolutionary tree, to highlight the evolution of the fusion event.

• The *T. brucei *proteins, **AAX79872 **and **EAN76725**, are non-homologous, reside on different chromosomes, and are annotated as "putative, DNA topoisomerase IB, large subunit" and "putative, DNA topoisomerase IB, small subunit", respectively. The region of alignment between **EDP05938 **and **AAX79872 **extends for 464 amino acids, and consists of two domains: a non-conserved hydrophilic N terminus, and a DNA-binding fragment (PF02919) (Figure 4A). The region of alignment between **EDP05938 **and **EAN76725 **extends for 73 amino acids, and coincides with the C-terminal catalytic core (PF01028) (Figure 4A). In all other eukaryotes, outside the kinetoplastids, DNA topoisomerase IB exists as a single fused protein (Figure 4B), and the apparent fission in *T. brucei *was previously identified by sequencing and protein purification, and experimentally verified by catalytic activity characterisation of the protein pair, as well as genetic studies of essentiality of the genes encoding both subunits [34,35]. Only the large subunit is conserved in bacteria, and neither the large nor the small subunit are found in the euryarchaeota (Figure 4B).

• The *T. brucei *proteins, **AAX79657 **and **EAN76651**, are non-homologous, reside on different chromosomes, and are annotated as "putative, electron transfer protein and "putative, succinate dehydrogenase", respectively. The region of alignment between **EDP08267 **and **AAX79657 **extends for 115 amino acids, and consists of the N-terminal half of the multi-domain succinate dehydrogenase/fumarate reductase iron-sulfur subunit (IPR00489, Figure 5A). The region of alignment between **EDP08267 **and **EAN76651 **extends for 92 amino acids, and consists of the C-terminal half of the multi domain succinate dehydrogenase/fumarate reductase iron-sulfur subunit (Figure 5A). Indeed, one of the *T. brucei *proteins, AAX79657, corresponds to the first half of the "succinate dehydrogenase iron-sulfur subunit of complex II", and the other, EAN76651, to the second half. Also both proteins are similar to, for example, protein SDH2 from *Schizosaccharomyces pombe *(P21911) which is annotated as "Succinate dehydrogenase [ubiquinone] iron-sulfur subunit, mitochondrial", with AAX79657 aligning to the first half and EAN76651 aligning with the second half. Interestingly, this protein binds two iron-sulfur clusters, one at 34-114 aa, and one at 155-185 aa, so in *T. brucei *these two domains are split between the two proteins. Succinate dehydrogenase (also known as complex II) is part of the mitochondrial electron transport chain for respiration/oxidative phosphorylation, which is generally thought to be inherited from the ancestor of mitochondria, i.e. from bacteria. So, in bacteria and most eukaryotes this protein is fused, but in trypanosomes it is split into two (Figure 5B). This split is conserved in other organisms similar to *T. brucei*, such as *T. cruzi *and *Leismania*. Succinate dehydrogenase (EC 1.3.5.1) was originally reported as not found in the *T. brucei *genome publication [14] (supplementary table ten). However, the perplexing result identified by our fusion analysis method was recently verified experimentally in a publication which identified all the components of complex II in *T. cruzi*, and which reported that SDH2 is encoded by two different proteins (XP_847169, and XP_826981, which correspond to AAX79657 and EAN76651, respectively) [36].

## Conclusions

Two polypeptides A and B in one organism, are likely to interact if their homologues are expressed as a single polypeptide AB in another. The *in silico *method used to detect such protein fusions is called domain fusion analysis and the composite polypeptide AB, is referred to as a Rosetta Stone protein, as it gives information about a functional link between domains A and B. The method was introduced by Marcotte *et al. *[1] and Enright *et al *[2], and is used to infer that two genes or proteins, which are not necessarily similar to each other, i.e. have different domain architectures, may interact, i.e. participate in the same biological pathway or be part of the same protein complex. It should be noted that the domain fusion analysis method often detects "promiscuous" or paralogous domains, which occur at a high frequency in many different protein sequences that do not share similar functions [10,12]. In the present study, one major goal was to exhaustively check the results to remove false positives, thereby increasing the robustness of predictions made using the Rosetta Stone analysis.

We analysed the proteome of *Trypanosoma brucei *against seven divergent protists. The automatic analysis, based on the BLASTp algorithm with appropriate thresholds for E-value, alignment length, protein coverage, and excluding homologous sequences, produced 81 putative composites, i.e. 3-38 fusion events detected in each species (Table 2). After verification by reverse BLAST, and by looking at the functional domain architecture, we rejected 85% of the putative composites. The remaining 12 results were analysed further by checking for conservation in the closely related species *Leishmania major*, after which only 5 composites were considered genuine. This result can be considered very conservative compared to previous analyses which for example, analysed 30 microbial genomes and detected 16-458 fusions in each [5]. This is most likely due to the more strict verification criteria used in the present study, including 70% protein coverage in alignments [13], reverse BLAST [37], and checking against the closely related species *L. major*. Indeed, the selectivity and the performance of our automatic in-house software were tested by comparing the same organisms analysed by Enright *et al. *[2]. Although almost 90% of the events reported by Enright *et al. *were also detected by our method (Additional file 1), only 20% of these conformed to all our selection criteria (%coverage, similarity, etc.). Many are classified by our software as paralogues, or participate in multiple fusions events indicative of promiscuous domains that are found in many modular proteins. However, our software also detected a number of events not reported by Enright *et al. *(Additional file 1) so, although it is selective, it is also highly sensitive. Nonetheless, the lower number of detected fusion events among the species analysed may be indicative of a lower occurrence of protein fusions or fissions within protists, as opposed to prokaryotes, yeast, and metazoa; further analysis including more species would be needed to verify this. The fact that most of the results of the automatic analysis did not pass our verification criteria, indicates a very high occurrence of false positives, and highlights the need for extra verification steps, especially reverse BLAST [37]. A 36.4% false positive rate was reported by Marcotte *et al. *[38] not including proteins annotated as "unknown". The extra verification step proposed here, checking for conservation of the fusion or fission event in a closely related species (in this case, *Leishmania major *for *Trypanosoma brucei*), is a generally applicable verification method that can be useful for other domain fusion analysis studies.

Of the five results analysed in detail, two have previously been independently identified and experimentally verified in *T. brucei*, namely the two fusion events identified in *Chlamydomonas *resulting in a split topoisomerase IB, and a split succinate dehydrogenase in *T. brucei *[34-36]. These provide direct experimental confirmation that the fusion-linked proteins are indeed part of the same complex and interact, confirming that the *in silico *analysis is finding functionally relevant pairs of genes. Both cases appear to result from a unique fission event in the kinetoplastida. The reason for this is unknown (it may be related to the notoriously unique nature of the trypanosome mitochondria, also known as kinetoplasts), but the fact that these heterodimeric proteins are unique to the kinetoplastids, marks them as potential drug targets for new therapeutic strategies [34,36]. Analysis of the topoisomerase IB homologues across prokaryotes and eukaryotes, favors a single fission event in trypanosomatids; an alternative scenario proposed by Bodley *et al. *[34], that the situation in trypanosomes represents the persistence of discrete proteins, and that the trypanosomes provide a missing link between a hypothesized ancient independent catalytic domain and the contemporary fused composites, is not supported by our analysis.

The fusion event identified in *Monosiga*, resulting in a combined "glutamine hydrolysing (not ammonia-dependent) carbamoyl phosphate synthase" (EC 6.3.5.5) and "aspartate carbamoyltransferase" (EC 2.1.3.2), appears to be common among fungi, metazoa and some bacteria, and concerns enzymes that participate in subsequent steps of the pyrimidine biosynthesis pathway. Heterotrimeric versions also exist in the bacteria, archaea, and archaeplastida, suggesting multiple independent fusion events between these domains. This is in agreement with previous domain fusion analyses that have identified many composites consisting of pairs of functionally linked proteins that participate in the same metabolic pathway [1,5,7,8].

Two of the results reported here are truly novel. One is the fusion in *Entamoeba *of a TBC GTPase activating domain with an N-terminal Ankyrin domain. This suggests that the two interact, and some support for this is lent by a recent study of GTPase activating proteins, that reported that TBC proteins do not directly interact with Rab proteins, but require another protein-interaction domain, confirmed by truncation mutant analysis for the case of a C-terminal Ankyrin domain [33]. Therefore, although the particular fusion is unique to the amoebozoa, further studies are needed to identify putative extra binding partners that mediate Rab and TBC protein interactions, and our results provide a testable starting point for such studies. As Rab activity is crucial to many cellular processes in trypanosomes [39], disruption of such interactions could provide a further therapeutic drug target. The other novel result is the fusion in *Phytophthora *of a hydroxymethylglutaryl-CoAlyase with a SNF7 domain protein. This fusion, which is also shared by diatoms and brown algae, may suggest a role for ubiquitylation or the ESCRT system in the sorting of hydroxymethylglutaryl-CoAlyase. Again, this is a testable hypothesis.

The functional links identified through domain fusion analysis in this study agree with the general principles for gene fusion and fission events established by previous studies. For example, gene fissions are relatively rare events compared to fusions [11], and in this case two lineage-specific fissions were identified in trypanosomes (Figure 4B, 5B), both of which have already been experimentally verified. The other gene fusions identified, are either lineage-specific (e.g. Figure 3B) or have occurred multiple times or been transferred by horizontal gene transfer (e.g. Figure 1B, 2B). Horizontal gene transfer has played an important role in the evolution of fused proteins [8]. Within the eubacterial and archaeal groups, often only domains of one component protein within a fusion-linked pair were identified, when the other is eukaryote-specific. Finally, as has been reported previously, the genes participating in fusions are not confined to a specific category or genomic position. The advantages of gene fission are that separate domains may permit the subunits to function independently, perhaps in conjunction with other partners, as well as the possibility of exchanging or replacing domains. The advantages of gene fusion are that protein-protein interactions are enhanced by proximity and do not need to rely on diffusion. The low number of fusion-linked gene pairs identified here may indicate a lower occurrence of this evolutionary mechanism within parasitic protozoa, but more data would be needed to reach a firm conclusion on this.

## Methods

### Proteome sequence retrieval

In the present study we compared seven protists, representing all eukaryotic lineages (Table 1), against the kinetoplastid protozoan *Trypanosoma brucei TREU927*. As our primary interest was the analysis of the proteome sequences, we chose organisms with completely sequenced and annotated genomes. Based on these criteria, we chose the following species: Unikonts-*Candida albicans, Monosiga brevicollis, Dictyostelium discoideum, Entamoeba histolytica*; Plantae-*Chlamydomonas reinhardtii*; Stramenopiles-*Phytopthora infestans*; Excavates-*Giardia intestinalis*. The complete proteomes of these parasites were retrieved form the NCBI genetic sequence databases and UniProt Knowledgebase [40,41].

### Identification of possible fusion links

**Figure 6:**
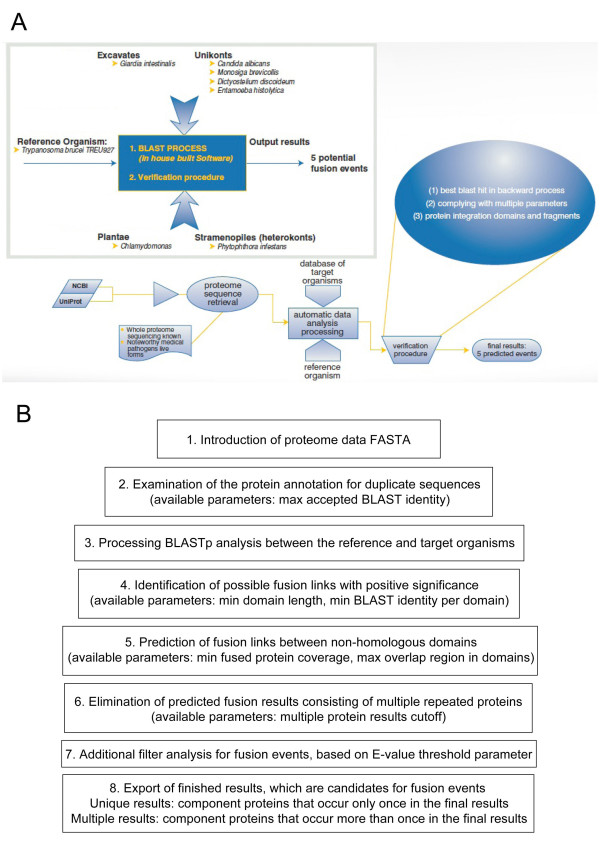
**Analysis workflow for identifying composite proteins**. (A) Work flowchart to determine gene fusions and functional linkages between *Trypanosoma brucei *and seven other protists. Amino acid sequences collected from NCBI and UniProt databases were used to identify fusion linked sequences by an automatic in-house built software module. The top scoring hits were verified by various criteria, such as the top scoring hit on the reverse BLAST process, the E-value threshold, the similarity and functionality of domain architecture between the component and composite proteins. (B) Outline of the software, with a focus on the parameters which can be altered by the user at various steps of the process.

We used a home-built software tool [42] based on the BLASTp search algorithm [43] to identify gene fusion links (Figure 6). The output of the program consists of pairs of non-homologous *T. brucei *proteins, which appear fusion-linked in other species, i.e. when queried against the proteome of another organism, both match the same protein as their best BLAST hit. We analysed the entire proteome of *Trypanosoma brucei TREU927 *against the entire proteome of each target organism. To parse the results and to avoid inaccuracies, we used the following settings for the automatic data analysis program:

• The software algorithm was developed to exclude any homologues which are 85% identical over the whole sequence length. Hence, a protein with a similarity value of 85% or higher to a larger protein of the **same**organism, was deleted from the proteome. On average, this removed about 7% of each proteome.

• A fusion-linked protein was taken into consideration only if each component had a minimum DOMAIN length of 70 amino acids, along with a minimum of 27% identity with the composite protein, based on the BLAST alignment per domain.

• We automatically excluded all results with an E-value higher than the threshold of 10^-3^, as not statistically significant.

• Two proteins were considered to be fusion-linked only if each of them aligned with a minimum PROTEIN coverage of 70% to the same reference sequence (based on the protein annotation of the target organism).

These parameters were chosen based on previous publications [2,13] and after fine-tuning, i.e. after iteratively checking the validity of preliminary results manually.

### Determining the significance of the predicted fusion results

To estimate the accuracy of the fusion links, we developed a manual verification pipeline:

1. The open reading frame of a putative fused protein in each target organism was split between the two BLAST hits, and we checked whether the two component parts returned the original two reference proteins of *Trypanosoma brucei TREU927 *as their best BLAST hits (reverse BLAST [37]).

2. To exclude misleading annotations, we looked for fusion links between *Trypanosoma brucei TREU927 *and *Leishmania major*, another parasitic kinetoplastid. Both organisms have similar genomes and their proteomes feature a high percentage similarity [14]. To examine whether the putative *T. brucei *fusion-linked proteins are homologous to each other, we repeated the BLASTp procedure using the *Leishmania major *proteome as target. If the *T. brucei *proteins matched the same protein in *L. major *they were considered homologous and excluded from further analysis.

3. We identified the domain architecture of the composite form of the fusion-linked proteins in the target organisms and checked for orthologous domains in their component (split) proteins.

Based on these three quality controls, the results were categorised into three groups:

• One group consists of composite proteins that have likely undergone fusion events, since (a) the split protein pair consists of two non-homologous proteins, and (b) the annotated interactions between the domains of the composite protein give a functionality equivalent to that of the domains of the split protein pair.

• Another group consists of proteins with domains of poorly characterised function, such as "unknown" or "general function"; fusion analysis can be used to predict the function of an unknown protein if it is fusion-linked to a partner of known function.

• The third group contains results where the split protein pair in *Trypanosome brucei *consists of homologous proteins. The links between homologous multi-domain proteins were not analysed further.

### Examination of the evolution of fusion links

The tree of life is an ever-evolving depiction of life's common ancestry. We used a tree of life, previously designed by the *Smithsonian Institution *([44] with permission), that has been used to illustrate the evolutionary relationship of humans to other common eukaryotes, as well as to the eubacteria and archaea. The tree was adapted to map our results for the identification of fusion and fission events. Thus, for each putative fusion linked protein pair identified, we examined whether these predicted proteins most likely occurred through protein fusion or fission. To calculate fusion or fission results we used the sequence pairs identified in *T. brucei *as fusion-linked (see above) and searched for homologues against all the available sequences in the NCBI database of all organism family groups shown in the tree. Within each organism family group, we selected the BLAST hit with the highest identity value and the lowest E-value threshold, as a single representative orthologue. The generated results were classified into 5 classes, based on whether the *T. brucei *fusion linked protein pair retrieved:

• a single protein composed of two regions corresponding to the *T. brucei *proteins (**fused**)

• two proteins, each one corresponding to one of the *T. brucei *proteins (**heterodimeric**)

• three proteins, one corresponding to one of the *T. brucei *proteins, and the other two corresponding to the second *T. brucei *protein (**heterotrimeric**)

• a single protein corresponding to only one of the *T. brucei *proteins (i.e. one part of the composite was not found, for example, in Figure 4B, bacteria retain only the DNA topoisomerase IB large subunit)

• proteins with low sequence identity **(data not available/not found)**. This may be because the whole proteome of each family group within the tree of life might not be complete or available through the NCBI or UniProt databases.

In addition, we attempted to elucidate the biological function and the metabolic pathways of the fusion linked proteins. We were able to claim that the evolutionary scenario of fused and fission domain architecture is valid, only in cases where the characteristics or the function of the fusion and fission events were similar to those of the reference protein.

## Competing interests

The authors declare that they have no competing interests.

## Authors' contributions

SK conceived and supervised the project and provided funding. DD and PT carried out the analysis, and VLK helped with data interpretation. VLK, DD and PT drafted the manuscript and figures and SK helped with critical revision; all authors read and approved the final manuscript.

## Supplementary Material

Additional file 1 **Comparison of our results with those of Enright *et al***. [2]. To test the selectivity and performance of our automatic in-house software we used it to analyse the proteomes of the same organisms analysed by Enright *et al. *[2]. The table (Worksheet "COMPARISON") is based on supplementary table one of Enright *et al. *[2], showing "the 64 fusion events in the genomes of *E. coli, H. influenzae *and *M. jannaschii*, detected on the basis of composite proteins in these three genomes plus the genome of *S. cerevisiae*. Columns COMPONENT list the component gene/protein names (or identifiers); column COMPOSITE lists the composite (fusion) gene/protein name (or identifier); columns with species names list the gene identifiers from the corresponding species; N lists the maximum number of possible pairwise interactions; COMMENT includes various comments for specific cases. White cells in the species columns contain component proteins and blue cells contain composite proteins, on the basis of which interactions are predicted. The sort order follows the three species against the composite-protein sequence-identifiers for the yeast genome, and then the other three species in succession. Genes are named when different names are used; where no name is available, the sequence-identifier is used instead. '#" indicates the absence of a component from a multiple fusion event (case 8). Interacting pairs are separated by "/", while paralogues are separated by commas." A column next to each species name shows the results from the present analysis, where "-" refers to the events not identified by our software (12.5% of the total) but which might be genuine, "c" refers to the events not identified by our software which are probably artefactual in the original analysis (13.5% of the total; details for each result are given in the "comments" columns), "p" refers to paralogues (5.7%), "m" to matches i.e. domains that participate in multiple interactions (34%), "d" to doubles i.e. domains that are involved in two separate fusions events (12.5% of the total), and "u" to unique results that conform to all the selection criteria (21.5% of the total). The percentages (categories) of results from our analysis are shown at the end of the table along with comments concerning each fusion event and the reasons for any difference in the results. In total, we can confirm 87,5% of the 88 fusion events reported by Enright *et al. *In addition, our software identified a further 27 events not reported by Enright *et al. *(Worksheet "UNIQUE"). Click here for file

Additional file 2 **Alignments of the fused proteins identified in different species, with the corresponding split protein pairs in *T. brucei***. A: BLAST output alignment of the *P. infestans *EEY58132 composite protein with the *T. brucei *proteins AAX79027 and AAX70704. B: BLAST output alignment of the *M. brevicollis *EDQ88211 composite protein with the *T. brucei *proteins AAX70833 and AAX70835. C: BLAST output alignment of the *E. histolytica *EAL47672 composite protein with the *T. brucei *proteins EAN76787 and EAN78273. D: BLAST output alignment of the *C. reinhardtii *EDP05938 composite protein with the *T. brucei *proteins AAX79872 and EAN76725. E: BLAST output alignment of the *C. reinhardtii *EDP08267composite protein with the *T. brucei *proteins AAX79657 and EAN76651. Click here for file
