# Supplementary material for: Protein functional links in Trypanosoma brucei, identified by gene fusion analysis
Source: BMC Evol Biol. 2011 Jul 5;11:193. doi: 10.1186/1471-2148-11-193 (PMC3155505; doi:10.1186/1471-2148-11-193)
Supplement: Additional file 2 — Alignments of the fused proteins identified in different species, with the corresponding split protein pairs in T. brucei. A: BLAST output alignment of the P. infestans EEY58132 composite protein with the T. brucei proteins AAX79027 and AAX70704. B: BLAST output alignment of the M. brevicollis EDQ88211 composite protein with the T. brucei proteins AAX70833 and AAX70835. C: BLAST output alignment of the E. histolytica EAL47672 composite protein with the T. brucei proteins EAN76787 and EAN78273. D: BLAST output alignment of the C. reinhardtii EDP05938 composite protein with the T. brucei proteins AAX79872 and EAN76725. E: BLAST output alignment of the C. reinhardtii EDP08267composite protein with the T. brucei proteins AAX79657 and EAN76651. [file 1471-2148-11-193-S2.PDF]

```
Query= EEY58132 Phytophthora infestans hydroxymethylglutaryl-CoA lyase, mitochondrial precursor

> AAX80809 hydroxymethylglutaryl-CoA lyase, putative;
           3-hydroxy-3-methylglutarate-CoA lyase, putative
           Length = 431

 Score =  115 bits (289), Expect = 2e-026,   Method: Compositional matrix adjust.
 Identities = 82/284 (28%), Positives = 127/284 (44%), Gaps = 25/284 (8%)

Query: 15  SFVKIVEVGPRDGLQNEKTNVSTDDKVKFINLLSETGLSAIEATSFVSPKWVPQMADNAY 74
           S +++VE  PRD +Q     + T+ K++++  L + G  A++  SFVSP+ VPQM D+
Sbjct: 9   SSIRMVEC-PRDAMQGLPHFIPTEQKIRYLKALLKCGFYALDCGSFVSPRAVPQMRDSTE 67
Query: 75  VLKGISR-----KQGVSYPVLTPNIKGFDSAV-AAGAEEVAIFGAASEAFSQKNINCSIE 128
           V+    +     K      V+  ++ GF  A+   G   +       E F Q+N   SI
Sbjct: 68  VIANCWKTMQEEKAAPKLSVVVASLAGFKQALETPGVSVIGYPIGCCERFQQRNAKKSIA 127
Query: 129 ESLERFR----------------PVCEKANSLGIRVRGYVSCVLGCPYQGPVDPSAVATV 172
            SL+  R                PV    +  G  +  Y+S   G PY        V  +
Sbjct: 128 MSLDEIRNIKEATDAFNAQRSSNPVAPNEDVNGRELLIYISMAFGNPYGESHSIDLVEKL 187
Query: 173 ALKMLEMGCYEVSLGDTIGVGNPASTLAMLQATKQVIPAERLAVHFHDTYGQALSNILIA 232
             +++  G  ++SL DT GV  P          ++  P    A HFH    +A   I+ A
Sbjct: 188 VGELVASGARDISLADTTGVAQPPLIFDTFTQLRKKFPDVTFAGHFHSNAVEARGKIVAA 247
Query: 233 LQEGIAVVDSSAAGLGGCPYA--SGASGNVATEDVLYMVHGLGI 274
           L  G  ++DS+  G+GGCP+A   G  GNVATE V+   +  G+
Sbjct: 248 LDAGCTMIDSALCGMGGCPFAKDDGLVGNVATEVVVKALEERGV 291

> AAX70704 hypothetical protein, conserved
           Length = 215

 Score = 59.7 bits (143), Expect = 2e-009,   Method: Compositional matrix adjust.
 Identities = 50/154 (32%), Positives = 79/154 (51%)

Query: 355 AQITSKDKAILDLKNARDRLKKYQSRLDIEANQLHDSAKRLLQAGKRDRAKLALKLKKYK 414
           A ITS D+  L+LK  RD++     + +  A   H  A  LL+ GKR+ A   LK +K +
Sbjct: 21  AVITSLDRVQLELKLQRDKIMAAIRKYERAAEGEHVRAAELLRNGKRELALYCLKRRKAQ 80
Query: 415 EQQMHQADEHLIQVLGMLDTVEWETQQLQVFEGLKAGNSILNAIHKEMSVEAVEELMLET 474
             Q+      L  V  ++ TVE+   + +V E LK G   L+ ++  ++++ V ELM  T
Sbjct: 81  MSQITLVTSMLENVERLICTVEFAQIEREVVEALKCGKDELSKLNAILNMDDVLELMDST 140
Query: 475 EEAQVTANEISRIIGGNLTVEDEDAVLSELAEIE 508
            +    + +I+ I+   L V DE  +LSEL   E
Sbjct: 141 ADVVEESKQINEILAQQLVVYDESELLSELCSAE 174


--------------------
```

```
Query= EDQ88211 Monosiga brevicollis MX1 predicted protein

> AAX70833 glutamine hydrolysing (not ammonia-dependent) carbomoyl phosphate synthase,
putative
                  Length = 1833

 Score = 1150 bits (2975), Expect = 0.0,   Method: Compositional matrix adjust.
 Identities = 611/1235 (49%), Positives = 796/1235 (64%), Gaps = 62/1235 (5%)

Query: 1    MCDSTIKSLQKVLQNEGDAIKPVFGICLGHQLLSIAIGAKTYKLTFGNRGHNQPCSNNQS 60
            +C  TI++++  L  E    KP+FG+C+G+ +L++A G  TYK+ FG+RG NQP ++NQ
Sbjct: 231  LCTKTIENVRWALTQE----KPIFGVCMGNHMLALAAGGTTYKMKFGHRGQNQPSTSNQD 286
Query: 61   GRCYITSQNHGFATDASALPAG-WVPIFTNENDSTNEGIAHESLPFFSVQFHPEATAGPE 119
            GR  IT+QNHGFA D  +LP G W   F N ND  NEG+ H + PF SVQFHPE   GP+
Sbjct: 287  GRVVITTQNHGFAVDFKSLPQGDWEEYFFNPNDQCNEGLRHRTKPFSSVQFHPEGCCGPQ 346
Query: 120  DLELLFDVFLMQCRDKAARAQPLPQTIADFLIEKSYGKPSPPPATLPTKVVVLGSGGLTI 179
            D E LF  F+    D+  R++   +  A F               P KV+VLG+GG+ I
Sbjct: 347  DTEYLFGEFI----DQVKRSKT--KLAAQFK---------------PRKVLVLGAGGIVI 385
Query: 180  GQAGEFDYSGSQAIKALKEEGVQTVLINPNIATVQTMKGLADKVYFLPVTPEYVEQVIAY 239
             QAGEFDYSGSQ +KAL+EEGV+++L+NPNIATVQT   +AD+VYF+PVTPE VE+VI
Sbjct: 386  AQAGEFDYSGSQCLKALREEGVKSILVNPNIATVQTDDEMADQVYFVPVTPEAVERVIEK 445
Query: 240  ERPDGLLLAFGGQTALNCGVELYEKGIFEKYSVRVLGTPVQAIVDTEDREIFSQKLSQID 299
            ERPDG++L +GGQTALNCG++L + G+ +KY+V+VLGT +  I  TEDRE+F   L QI+
Sbjct: 446  ERPDGIMLGWGGQTALNCGLQLDKLGVLKKYNVQVLGTAISTITVTEDRELFRNALLQIN 505
Query: 300  EKCCPNIACTTVDEVVAAAEKIGYPVMMRSSFALGGLGSGVVDNVDDLREKARVALNNTT 359
            E    ++A T+V E + AA  IG+P+M+R++F LGG GSG+V++ ++L  K  VAL
Sbjct: 506  EPVAKSVAVTSVAEALKAAADIGFPMMVRAAFCLGGQGSGIVNSEEELSNKVEVALTVAP 565
Query: 360  QVMLERSLHGWKEIEYEVVRDCEDNTITVCNMENFDPLGIHTGESIVIAPSQTLSNDEYN 419
            QV+LE S+ GWKEIEYE+VRD  DN ITVCNMENFDP+G+HTGESIV+APSQTL+N+EY+
Sbjct: 566  QVLLEESVAGWKEIEYEIVRDIHDNCITVCNMENFDPMGVHTGESIVVAPSQTLTNEEYH 625
Query: 420  MLRTTAVKVVRHIGIVGECNIQYALNPFSREYYIVEVNARLSRSSALASKATGYPLAYVA 479
            MLRT A+K++RH+G+VGECNIQY L P SR Y ++EVNARLSRSSALASKATGYPLA+VA
Sbjct: 626  MLRTAAIKIIRHLGVVGECNIQYGLEPHSRRYVVIEVNARLSRSSALASKATGYPLAHVA 685
Query: 480  AKLALGHTLPKLRNSVTRKTTACFEPSLDYCVVKIPRWDLSKFPTVDKRLGTSMKSVGEV 539
             K+ALG  L +++N VT+ T ACFEPSLDY  VK PRWD++KF  V + +G+ MKSVGEV
Sbjct: 686  TKIALGKGLFEIKNGVTKTTMACFEPSLDYVTVKAPRWDVAKFNMVSQEIGSMMKSVGEV 745
Query: 540  MAIGRIFEEAFQKALRMVDLGFMGF-------EAGKVEINQEELVHGSEQRPFMLATALK 592
            MAIGR FEEA QKALRMVD    GF       E G         L   +  R F +  ALK
Sbjct: 746  MAIGRTFEEAVQKALRMVDPSNNGFDTPKRLAEMGDKWDYMRALRVPTPDRIFAICRALK 805
Query: 593  RGYTVDQLYDLTKIDKWFLYRLQRIV-MHGDLLKQY--DMHSVPAPILREAKQLGFSDAH 649
             G TVD+++ LT+IDK+FL +LQ ++ M  +L   Y   + ++    L   K  GFSD
Sbjct: 806  EGITVDEIHRLTRIDKFFLNKLQLLIEMQRELTTLYRGKLDTITYDHLLAMKAHGFSDVQ 865
Query: 650  IGAIVNGNEVGVRTRRQTLNILPVVKQIDTVSAEYP-AYTNYLYLTYNGTANDVEFEGDY 708
            I    +       VR RR  LNI P VKQIDTV+ EYP A   YLY TYN    +DVEF
Sbjct: 866  IAEYLQCTTDDVRKRRYKLNITPKVKQIDTVAGEYPAAQCCYLYTTYNAQHDDVEFNDRM 925
Query: 709  VMVLGSGVYRIGSSVEFDYCAVGCVRELRRLGFKTIMVNYNPETVSTDYDECDRLYFDQL 768
              VLG GVYRIGSSVEFDY  V   RELRRLG K I++NYNPETVSTDYDECDRLYF+++
Sbjct: 926  YAVLGCGVYRIGSSVEFDYGGVLVARELRRLGNKVILINYNPETVSTDYDECDRLYFEEV 985
Query: 769  TFETVMDIYELETARGIVLSMGGQIPNNIANALQRANAKVLGTTPDMIDNAENRYKFSRM 828
            + ETV+DI   E   G+++S+GGQI  N+A  L+      +LGT P  +D AE+R KFS+M
Sbjct: 986  SEETVLDILLKEKISGVIISLGGQIVQNMALRLKEHGLPILGTDPVNVDKAEDRNKFSKM 1045
Query: 829  CDSIGVDQPEWKELTSVESAKAFAEKVSYPCLMRPSYILSGTAMRVAHSAHDLENDFRTA 888
            CD +GV QPEW   TSV+   AF ++V +P L+RPSY+LSG+AM V  S  D++     A
Sbjct: 1046 CDQLGVPQPEWILSTSVQDVHAFCQRVGFPTLVRPSYVLSGSAMAVISSPEDIDRYLTKA 1105
Query: 889  VVVSRDYPVVLSKFILDAKEIEVDAVASNGELVASCISEHVENAGVHSGDATIVHPPVDL 948
             +VS  +PVV+SK+   A E +VD VA +G ++   I EH+ENAGVHSGDAT+  PP
Sbjct: 1106 SLVSGTHPVVVSKYYEGAMEYDVDIVAHHGRVLCYAICEHLENAGVHSGDATMFLPPQHT 1165
Query: 949  TQKTMNGCLAIAGKIAKALHITGPFNIQF-IAKDDNLKVIETNVRASRSFPFVSKTANVD 1007
             ++ M       A +IA  L + GP N+QF + KD+ L+VIE N+R+SRS PFVSKT  +
Sbjct: 1166 KKEVMKRIYEAATQIAGELDVVGPMNVQFLLTKDEQLRVIEANIRSSRSVPFVSKTLGIS 1225
Query: 1008 MIAIATRVMLGL-DPEVPP--QVKPDRVGVKVPQFSFNRLAGADPTLGVDMISTGEVACF 1064
```

```
                A+     +L    D E+ P   + K    +G K P FSFNRLAGADP LGV+M STGE+  F
Sbjct: 1226 FPAVMVSALLSRPDSELVPIRRAKMTHIGCKAPMFSFNRLAGADPILGVEMASTGEIGVF 1285
Query: 1065 GKTREEAYLKAMISTSFRLPRPQGNVLLSIGGYDGKLEFLDSTRRLQQLGFKLFGSMGTA 1124
             G  + E + KAM+  +FR    P   V +S          E L    ++ +    LF S  T
Sbjct: 1286 GCDKREVFRKAMLCQNFRY--PTKGVFISSDVDAVTEELLPHLEKISKT-LPLFASTHTG 1342
Query: 1125 DFYLSHGVEIKPVDWLADTEDIESIKDNLLDGHYDMVINLPMRNKYR-----------RP 1173
                  HG+     +     +  D  + +  L    +D+VI L  RNK +             P
Sbjct: 1343 AVLTKHGIPHTVLTQRHEDGDNPTYEVELAARRFDLVIQL--RNKRKDFILRSCTRENAP 1400
Query: 1174 ASYMTSGSLARRMAVESKVPLITNIKCAKLFIAAL 1208
               Y        RR+AV+   V L+T      K+F   L
Sbjct: 1401 PDYW-----VRRLAVDYNVALLTEPNVVKMFCETL 1430
```

>AAX70835 aspartate carbamoyltransferase, putative [1191671 - 1192654] MW:35910.64 Length = 327

```
 Score =  289 bits (740), Expect = 4e-078,   Method: Compositional matrix adjust.
 Identities = 157/325 (48%), Positives = 216/325 (66%), Gaps = 19/325 (5%)

Query: 1613 LAPPSILKGRNILSVEQFGKRELHALFNRAHEFHSTKGAS---KLLDGKVMANIFYEPST 1669
             L P + LKG++I++   QF + ++ AL    A         A     + L+G++M   +F+E S+
Sbjct: 4    LQPVTSLKGKSIITAAQFTRPDIDALIRLATALKEKICAGEVLRFLEGRIMTPLFFEDSS 63
Query: 1670 RTMCSFASAMQRLGGTVITLSDMKSTSVSKGESLSDTVRTMECYTDVIVLRHPAKGAMRE 1729
             RT+ SF +AM RLGG V+      +++SV+KGE+L DTVRT++ Y+DV+VLRHP + A+ E
Sbjct: 64   RTLNSFCAAMARLGGRVVYFK-AETSSVNKGETLGDTVRTLDSYSDVLVLRHPKQEAITE 122
Query: 1730 ASELLRRPLINAGDGVGEHPTQALLDVFTIREELGTVNGLHIAMVGDLKNGRTVHSLARL 1789
             A       P++NAG+G GEHPTQALLDV TI  ELG V+G  IAM+GDLK GRTVHSL +L
Sbjct: 123  AVAKATHPVLNAGNGAGEHPTQALLDVLTIHSELGRVDGSTIAMIGDLKMGRTVHSLLKL 182
Query: 1790 LSR--YNVTLHYISPPSLAMPDDVVEFVK----ASGITQHTHASI-EEIVADIDVLYVTR 1842
             L R      T+ +++P +L MP DVV+ +K     ASG+T   +  S+ EEI+   DVLY TR
Sbjct: 183  LVRNFKMKTIFFVAPDALQMPQDVVDSLKQEIAASGVTIRSSNSLTEEILGQCDVLYATR 242
Query: 1843 VQKERFEDEAE--------YERVKNELVVTPETLINAKKRMIVMHPLPRVNEISPDVDTD 1894
             +QKERF   A          +E  K ++V+   E +  AK +MIVMHPLPR +E+   VD D
Sbjct: 243  LQKERFAAAALDEAKALQAFEAAKADIVINAERMKKAKAKMIVMHPLPRNDELCTSVDED 302
Query: 1895 PRAAYFRQMEYGVSVRMALLASVLG 1919
             PRAAYFRQM+YG+ +RMA+L SVL
Sbjct: 303  PRAAYFRQMQYGMYMRMAILYSVLA 327
```

Query= EAL47672 Rab GTPase activating protein, putative

\> EAN78273 GTPase activating protein, putative
Length = 418

Score = 107 bits (268), Expect = 6e-024,   Method:Compositional matrix adjust.
Identities = 96/335 (28%), Positives = 156/335 (46%), Gaps = 38/335 (11%)

```
Query: 303 TDEFGYVKDQTQ---TEDEIKKITSQE---REKKWEIMLDEWNKSGKVPRA-LFHRVYKF 355
             DEFG++ D+ +    +  I+ I  +    RE KW+ M   W    K  R+ +  R  K
Sbjct: 92  VDEFGFIIDEEEHDRVQRYIRGIDGKRVARREVKWQKMQANWESMSKKRRSKVKSRCRKG 151

Query: 356 VPLNKRKKYWEIELKINQSIQEYN----GYF--ARITELPERGKDDEQIHKDVMRAHQNN 409
            +P + R   W+  L I   ++  N    G +   R+ ++ + G     I +D+ R    +
Sbjct: 152 IPSSFRGAAWQ--LLIGSYLEMLNPGNEGTYDCLRLKDISDEGLKG-TISRDLPRTFPKH 208

Query: 410 IHFMAKFNDGQRTLFRVLRAWSMQD-DLGYVQGMSDLCGFLILILQEEEEVYWGFSALMN 468
            + F  +   GQ  L  VL A++  D ++GYVQGM+ + G L   + EEE  +W    LMN
Sbjct: 209 VLFREEGGIGQTFLRNVLHAYANIDPEVGYVQGMAFVVGALYTQMTEEE-TFWALHTLMN 267

Query: 469 NPKYNLREMFLPGFPGINKCAFVAMKVMKKYHPKIFYHLKSKEYDFDNWKTYMLEYFMLW 528
              KY LREM+ PGFP ++K  +   ++M K  P ++ H       F+    +   Y   W
Sbjct: 268 GEKYRLREMYKPGFPMLHKLFYQLQRLMAKLLPNLYEH-------FEELGVHPTYYASRW 320

Query: 529 FCRCFHPEFE----VRILDLILMEGWEIVF--SIFSAILHYSKVEILKMDEYIFIDKAL- 581
            F   F  +F     +RI D+ L EGW+I+F  +I    L   ++  +  +E IF  K L
Sbjct: 321 FMTLFVYDFNFRAVLRIWDIFLSEGWKIIFRIAIVLLKLEERRLLAMSFEEIIFATKTLE 380

Query: 582 --ADPMTLM----GSQFDQDKFINFVKKARITPKE 610
               DP  L+    G +F   +   F ++  +  +E
Sbjct: 381 QGKDPDELIRRAHGVRFKTAELEAFAREYELNKRE 415
```

\> EAN76787 huntingtin interacting protein (HIP), putative
Length = 820

Score = 48.9 bits (115), Expect = 4e-006,   Method:Compositional matrix adjust.
Identities = 39/129 (30%), Positives = 57/129 (44%), Gaps = 16/129 (12%)

```
Query: 95  VKQLLAAGITKSAQDINGNNALIRGVIAGRTNACVYLARKMNKTGIDSQNNLGECAIYIA 154
           V   L  G   +A+D +G  AL   V  G   A V          +  +  LGEC
Sbjct: 58  VVTFLLGGADVNARDKDGATALHVAVTQGNDIATV--------CTLGREPFLGEC----- 104

Query: 155 CRRNNLKLLEKLIKYGGNVNIQNNKGITPLMVACFYMNLEMINLLIKAGANIDTVDSDKS 214
              +N  ++  LI  G ++N +N  G TPLMVA    N+  + LL++ GA I   D
Sbjct: 105 ---SNEMIIRFLIDNGADINARNASGETPLMVAAAKGNITAMRLLLERGAVITQRDDAGC 161

Query: 215 NCLHYMAKS 223
             LH+ ++S
Sbjct: 162 TVLHHASRS 170
```

```
Query= EDP05938 Chlamydomonas reinhardtii partial DNA topoisomerase I

>AAX79872 DNA topoisomerase IB, large subunit
          Length = 661

 Score =  432 bits (1112), Expect = e-122,   Method:Compositional matrix adjust.
 Identities = 232/467 (49%), Positives = 302/467 (64%), Gaps = 22/467 (4%)

Query: 23  SSKSGQVMWKTLKHAGVLFPPEYEPHGVKPLYDGKPVDLTPDQEEVATMFAVMKETDYMN 82
            ++K G+  W TL H GVLFPP Y PHG+  LY+ +  ++TP++EEVATMFAV++E DY
Sbjct: 66  TTKKGEKRWDTLLHNGVLFPPAYVPHGIPILYNQQKFEMTPEEEEVATMFAVLREHDYYR 125
Query: 83  KKVFLDNFWEGFKEVLGK-GHVIKDLKKCDFTPIYDWHMAQREAKKGISKEEKDRIKKEK 141
             +VF  NF++ ++E+L K  H I+ L+ CDF+ IY+WH  + E +K  ++EEK  +K+
Sbjct: 126 NEVFRRNFFQSWREILDKRKHPIRCLELCDFSAIYEWHQREVEKRKSRTREEKKELKRIA 185
Query: 142 DEKEAKYKVAYVDGRPEPVGNFRVEPPGLFRGRGEHPKMGKIKKRVYPRDITINIGENEP 201
            DE+   YK    +G+ E V NFRVEPPGLFRGRGEHP  GK+KKR+ P D+ +NIG+  P
Sbjct: 186 DEEAEPYKWCIWNGKKEQVANFRVEPPGLFRGRGEHPMRGKLKKRILPEDVVLNIGKEAP 245
Query: 202 VPEHPYPGQTWKEVKHDHTVTWLAYWKDTISTKDYKYVFLGATSTFKADSDLAKYEKARK 261
            +P+ P  G  WK V HD  VTWLA W +  +    KYV L  +ST K  SD AK+E AR+
Sbjct: 246 IPQAP-AGHKWKGVVHDQNVTWLAMWYEP-TIGQCKYVMLAPSSTLKGQSDYAKFETARE 303
Query: 262 LKEIIVDVRKNYERDWDSSDQRKRQMGVAMYFIDKLALRAGHEKDEDEADTVGCCTLKVE 321
            LK  I D+R++Y +D+ S+D+ +RQ  VA YFIDKLALR GHEK E+EADTVGCC+L+ E
Sbjct: 304 LKNHIDDIRESYTKDFSSTDEMERQRAVATYFIDKLALRVGHEKGEEEADTVGCCSLRKE 363
Query: 322 NIEIMGDNKVKFDFLGKDSIRYENIVEVDPRVYKNLEKF-KRIDHTGKRKQQGDQLFETF 380
            +IE+  DN V+FDFLGKDSIRY N V V P VYK L  F KR D          ++F
Sbjct: 364 HIELRPDNVVRFDFLGKDSIRYVNEVTVLPEVYKLLGSFIKRTD---------SEIFRKV 414
Query: 381 DAQDLNKELKNIMDGLSVKVFRTYNASIVLDRLLSEWEATKKGHSTAQTVDQKKVDYDIA 440
                LN  LK+ +  LS KVFRTYNASI LD    EW   K     A    D K V ++ A
Sbjct: 415 TPTTLNNYLKSFLKDLSAKVFRTYNASITLD----EWFREKPVDPKASLSD-KLVYFNKA 469
Query: 441 NKEVAILCNHQRSVPKTHTNQMEKIQEKLAGMNKELAELEDELRAAQ 487
            N EVA LCNHQRS+PKT    ++ I+ KL  + + +    D LR AQ
Sbjct: 470 NTEVAKLCNHQRSIPKTFHVSVQSIKYKLEDIKRTI----DTLRRAQ 512

>EAN76725 DNA topoisomerase type IB small subunit
          Length = 273

 Score = 71.6 bits (174), Expect = 5e-013,   Method:Compositional matrix adjust.
 Identities = 32/74 (43%), Positives = 49/74 (66%)

Query: 505 LPWALNSKIDKKKQAIAKVELQARSKEDLKTVALGTSKINYMDPRITVAWCKRNEVPIEK 564
             P  + ++I K ++ +  VE   + K+D KTV+LGTSK+NY+DPRI  +W   N VPI +
Sbjct: 193 FPRDIEAEIGKHEKRLKDVENMLKMKQDNKTVSLGTSKVNYIDPRIVCSWANENNVPISR 252
Query: 565 IFNKSLLAKFNWAM 578
            +F+ +L  KF WA+
Sbjct: 253 LFSATLQKKFPWAL 266
```

```
Query= EDP08267  Iron-sulfur subunit of mitochondrialsuccinate dehydrogenase


> AAX79657  electron transfer protein, putative
           Length = 242

 Score = 98.2 bits (243), Expect = 2e-021,   Method: Compositional matrix adjust.
 Identities = 48/116 (41%), Positives = 77/116 (66%), Gaps = 6/116 (5%)

Query: 60   FQIYRWNPDSDEKPKYASYQVDINNCGPMMLDVLLKIKDEQDQTLSLRRSCREGICGSCA 119
              ++ R++P+++ K +   SY+ D ++    M+LD++   +K  QD TL+ R SC EG+CGSCA
Sbjct: 30   LRLIRFDPETN-KQRVESYEYDKHH-DYMVLDLITAVKAHQDPTLAFRASCCEGVCGSCA 87

Query: 120 MNIDGSNTLACLCKVNRDPGHVGKVAPLPHMFVVKDLVVDMANFYAQYKSIKPYLQ 175
           MNI+G N+LAC+          V  V PLP+  V+KD VVD+ +F+ QY  I+P+++
Sbjct: 88   MNINGVNSLACITF----SQQVTTVGPLPNFPVIKDFVVDLRHFFRQYAYIRPFVR 139

> EAN76651  succinate dehydrogenase, putative
           Length = 188

 Score =  108 bits (269), Expect = 2e-024,   Method: Compositional matrix adjust.
 Identities = 46/93 (49%), Positives = 62/93 (66%)

Query: 191 SRAKLDGLYECILCACCSTSCPSYWWNSDKYLGPAVLLAAYRWIIDSRDDMTSERMKEVD 250
           +R +L+GLYEC+LCA C+ SCP YWWN + +LGPAVLL +YRW+I+  D     R+K  +
Sbjct: 74   NRNRLEGLYECVLCASCTGSCPQYWWNRELFLGPAVLLQSYRWLIEPLDRDFDSRVKMFE 133
Query: 251 DAYKLYRCKTIMNCATVCPKGLNPGKAINKIKQ 283
               +  C  I NC+  CPK LNPG A  +IK+
Sbjct: 134 HGPLVNFCHNIFNCSITCPKFLNPGMASKEIKR 166
```
